# Dynamic monitoring of circulating tumor DNA to predict prognosis and efficacy of adjuvant chemotherapy after resection of colorectal liver metastases

**DOI:** 10.7150/thno.59644

**Published:** 2021-05-12

**Authors:** De-Shen Wang, Hui Yang, Xiao-Yun Liu, Zhi-Gang Chen, Yun Wang, William Pat Fong, Ming-Tao Hu, Yuan-Chao Zheng, Yun Zheng, Bin-Kui Li, Yun-Fei Yuan, Gong Chen, Zhi-Zhong Pan, Lele Song, Yu-Hong Li, Rui-Hua Xu

**Affiliations:** 1 State Key Laboratory of Oncology in South China, Collaborative Innovation Center for Cancer Medicine, Sun Yat-sen University Cancer Center, Sun Yat-sen University, Guangzhou 510060, P. R. China.; 2 Research Unit of Precision Diagnosis and Treatment for Gastrointestinal Cancer, Chinese Academy of Medical Sciences, Guangzhou 510060, P. R. China.; 3 Department of Medical Oncology, Sun Yat-sen University Cancer Center, Guangzhou, People's Republic of China.; 4 Department of Molecular Diagnostics, Sun Yat-sen University Cancer Center, Guangzhou, People's Republic of China.; 5 HaploX Biotechnology, Co., Ltd., Shenzhen 518057, People's Republic of China.; 6 Department of Hepatobiliary Surgery, Sun Yat-sen University Cancer Center, Guangzhou, People's Republic of China.; 7 Department of Colorectal Surgery, Sun Yat-sen University Cancer Center, Guangzhou, People's Republic of China.

**Keywords:** colorectal liver metastases, prognosis, ctDNA, next-generation sequencing, adjuvant chemotherapy

## Abstract

**Rationale:** Hepatectomy and adjuvant chemotherapy after resection of colorectal liver metastases (CRLM) may improve survival, however, patients which may benefit cannot currently be identified. Postoperative circulating tumor DNA (ctDNA) analysis can detect minimal residual disease (MRD) and predict the prognosis and efficacy of adjuvant chemotherapy. Our study aims to determine the impact of serial ctDNA analysis to predict the outcome among patients undergoing resection of CRLM.

**Methods:** Between May 2018 and October 2019, 91 CRLM patients were prospectively enrolled. Whole exome sequencing was performed in 50 primary and 48 metastatic liver tissues. Targeted sequencing of 451 cancer relevant genes was performed in 50 baseline plasma to determine plasma-tissue concordance. We prospectively investigated changes in the amount and constitution of ctDNA in 271 serial plasma samples taken at different time points (baseline, pre-operation, post-operation, post-operative adjuvant chemotherapy (post-ACT) and recurrence) during the treatment of CRLM.

**Results:** Detected molecular alterations were highly consistent among baseline ctDNA, primary and liver metastases tissue. Patients with a higher variant allele frequency (VAF) level at baseline ctDNA represent a higher tumor burden, and decreased ctDNA during pre-operative chemotherapy predicted better tumor response. Patients with detectable post-operative and post-ACT ctDNA were associated with significantly shorter recurrence-free survival (RFS). ROC analysis showed that post-ACT ctDNA status was superior to post-operative ctDNA status in predicting RFS with an AUROC of 0.79. A significant difference in overall recurrence rate was observed in patients with detectable vs undetectable levels of ctDNA after resection of CRLM (79.4% vs 41.7%) and after completion of adjuvant chemotherapy (77.3% vs 40.7%). During adjuvant chemotherapy, patients with decreased ctDNA VAF after adjuvant chemotherapy had a recurrence rate of 63.6%, compared to 92.3% in patients with increased ctDNA VAF.

**Conclusions:** We envision that dynamic ctDNA analysis, especially in a post-ACT setting, might be used to not only reflect MRD but also to determine rational personalized adjuvant therapy after the resection of CRLM.

## Introduction

Hepatectomy remains the main potential curative treatment for long term survival in patients with colorectal liver metastases (CRLM) 1. However, up to 60% of patients will experience recurrence, a striking 30% of patients experience early recurrence and consequently reduced survival rate. Therefore, accurate prognostic markers are needed to achieve better prognostic prediction and help personalize patient treatment. Several scoring systems for prognosis have been developed to better identify patients who can benefit from CRLM resection. The most widely used system is the clinical risk score (CRS) proposed by Fong et al., which includes the number and size of metastases, primary tumor nodal status, preoperative CEA levels, and disease-free interval from primary to metastases <12 months 2. However, survival rate may vary considerably even among patients with the same CRS score, thus limiting its clinical value and acceptance 3. Currently, some molecular biologic markers are being used to guide therapy and prognostication in patients with CRLM. RAS mutations are found in 15-45% of colorectal cancer patients, and are associated with poor overall survival (OS) and recurrence-free survival (RFS) after liver resection 4-6. A previous study found that the addition of RAS mutation status to create a modified clinical score (m-CS) could outperform the t-CS in predicting survival after resection of CRLM 7. Furthermore, next-generation sequencing (NGS) has allowed the analysis of several genes with potential prognostic relevance to inform clinical decisions in CRLM beyond RAS. For instance, Lang et al. found that alterations of the SMAD family as well as the RAS/RAF pathway resulted in an extended clinical risk score (e-CS) that can effectively predict oncological outcomes 8.

In recent years, plasma circulating tumor DNA (ctDNA) has emerged as a promising non-invasive tool to repeatedly evaluate tumor burden and genomic profile 9, 10. Earlier studies have suggested that the post-operative detection of ctDNA ranged from 10-15% in patients with stage II and III colorectal cancer and nearly 50% in those who have undergone curative hepatectomy 11-13. In stage II and III colon and rectal cancer, several studies have reported that ctDNA testing can identify the minimal residual disease (MRD) after radical resection, thus identifying patients with the highest risk of recurrence 11, 14 and predicting the benefit of adjuvant treatment 15. ctDNA detection can also be used for the real-time monitoring of genomic abnormalities during anti-EGFR or anti-HER2 therapy in metastatic colorectal cancer patients, identifying clinically actionable resistance mechanisms and thereby guiding subsequent treatment 16-18. In addition, in patients with CRLM, limited studies have reported that pre-operative ctDNA detection could be used to select patients eligible for liver metastasectomy 19, 20. However, the sample size for ctDNA testing in these studies was small and only available during and after surgery.

Based on the clinical translational potential of ctDNA for prognosis prediction, molecular profiling, and disease monitoring in patients with CRLM, this study aims to elucidate the clinical value of serial ctDNA analysis in predicting the clinical outcome of patients during CRLM therapy.

## Patients and methods

### Patient's enrollment

Consecutive patients with CRC liver metastases were recruited between May 2018 and October 2019 at Sun Yat-sen University Cancer Center in China. The eligibility criteria were as follows: 1) pathologically and radiologically diagnosed with CRLM; 2) underwent liver metastasectomy with curative intent; 3) presence of tissue samples and, or blood samples for analysis. The exclusion criteria included those with 1) unresectable hepatic or extrahepatic metastasis; 2) at least one blood sample not available during and after treatment; 3) incomplete clinical information; 4) history of other cancers. The landscape and consistency of tumor tissue and baseline blood were analysed in patients who had both tumor tissue sample and baseline blood. The ctDNA VAF and prognostic value of serial ctDNA was analysed in all plasma specimen at each time point including baseline, pre-operation, post-operation and post-ACT. Association between decreased ctDNA VAF during the pre-operative chemotherapy and the tumor responses was analysed in patients who have both baseline ctDNA level at diagnosis, pre-operative ctDNA level and tumor response after neo-adjuvant chemotherapy, while the dynamic changes of ctDNA during adjuvant chemotherapy were analysed in patients who have both postoperative and post-ACT ctDNA level after adjuvant chemotherapy.

### Blood sample collection and DNA extraction

Blood samples were collected at diagnosis before treatment (baseline), before liver resection (pre-operation), after liver resection (post-operation), after completion of post-operative adjuvant chemotherapy (post-ACT) and in the event of disease progression (PD). For the extraction of ctDNA from plasma, 10 mL blood samples were collected in 10 mL K2 EDTA anticoagulant tube (BD biosciences, Franklin Lakes, NJ, USA) and centrifuged at 1600 × g for 10 minutes and at 4 °C. The supernatants were further centrifuged at 10,000 × g for 10 min at 4 °C, and plasma was harvested and stored at -80 °C until further use. ctDNA was extracted from 3-3.5 mL plasma using the QIAamp Circulating Nucleic Acid kit (Qiagen, Inc., Valencia, CA, USA) according to the manufacturers' instructions. For the extraction of genomic DNA from peripheral blood lymphocytes PBLs RelaxGene blood DNA system (Tiangen Biotech) was used, and the genomic DNA was preserved at -20 °C for further study. The quality control for DNA was achieved using Qubit 2.0 (Thermo Fisher Scientific), following the manufacturer's instructions. For the FFPE samples, ten 5 μm tissue sections were taken for DNA extraction, using the QIAamp DNA FFPE Kit (QIAGEN, Valencia, CA, USA), and following the manufacturer's instructions.

### Library construction, whole-exome sequencing, and data processing

The fragmented genomic DNA underwent end-repairing, A-tailing and ligation, and then was sequentially completed with indexed adapters, followed by size selection using Agencourt AMPure XP beads (Beckman Coulter Inc., Brea, CA, USA). The DNA fragments were used for library construction with the KAPA Library Preparation kit (Kapa Biosystems, Inc., Wilmington, MA, USA) according to the manufacturer's protocol. Seven to eight polymerase chain reaction (PCR) cycles, depending on the amount of DNA used, were performed on pre-capture ligation-mediated PCR (Pre‑LM‑PCR) Oligos (Kapa Biosystems, Inc.) in 50 μl reactions. The DNA sequencing of FFPE samples was performed using a WESPlus gene panel (an upgraded version of the standard whole-exome sequencing (WES), HaploX Biotechnology) for tumor tissue sequencing on the Illumina Novaseq 6000 system according to the manufacturer's instructions. The DNA sequencing of ctDNA samples was performed using a 451-gene NGS panel targeting the exome regions of 451 genes (HaploX Biotechnology). Sequencing using WESPlus was performed to an average depth of 500×, and sequencing using a 451-gene panel for ctDNA was performed to an average depth of 20000×.

Sequencing data were filtered by FastQC and aligned to the hg19 genome (GRch37) using Burrows-Wheeler Aligner (BWA). SAMtools was used to sort the BAM files and perform duplicate marking. The Glencore version 0.12.0 (https://github.com/OpenGene/gencore) was used to remove duplicate reads. Somatic variants were determined using MuTect2. A new panel of normal (PON) was created by in-house healthy individuals using GATK. ANNOVAR was performed to annotate the Variant Call Format file obtained in the previous step. A sample was defined as positive when variant allele frequency (VAF) ≥ 2% for WES Plus and ≥ 0.5% for the ctDNA 451-gene panel.

### Statistical analysis

Statistical analyses were performed using SPSS software, version 22.0 (SPSS Inc., Chicago, IL, USA) and R (version 3.4.4) for Windows. Assessment of statistical significance was performed using the Wilcoxon rank-sum and Fisher's exact test for categorical variables. The agreement between primary tissue and baseline ctDNA was assessed with McNemar's test. The Mann-Whitney U test was used to compare the VAF levels of different time points. RFS was the time interval from the date of liver resection to disease recurrence or the last date of follow-up. The Kaplan-Meier curves were used to calculate the survival rate, and the Log-rank test was used to compare them. Hazard ratios (HR) and 95% confidence intervals (CI) were calculated using univariate and multivariate Cox proportional hazards regression models to assess the survival impacts of the prognostic variables. The ROC curves were plotted by the R package “survival ROC”, and the prognostic factors of the 3-year RFS were calibrated by comparing predicted survival with observed survival. For all statistical tests, a *P* < 0.05 was considered statistically significant.

## Results

### Patient characteristics and sample collection

A total of 137 patients diagnosed with CRLM were enrolled, and 46 patients were excluded from this study because of unresectable hepatic or extrahepatic metastasis (n = 39) or consent withdrawal (n = 7) (Figure S1). Ninety-one patients received curative surgery of liver metastases, and the detailed clinical characteristics are presented in Table S1. The therapeutic regimen followed by these 91 CRLM patients was according to the ESMO guideline (2016), and neoadjuvant chemotherapy was an option based on surgery advances and prognostic oncological criteria which include the number of lesions and clinical risk score criteria. 20 patients had metachronous liver metastases, and 71 patients had synchronous liver metastases, among which 39 patients underwent simultaneous resection, and 32 patients underwent sequential resection. 84 patients (92.3%) were treated with pre-operative chemotherapy, and 83 patients (91.2%) received adjuvant chemotherapy after hepatectomy. The follow-up endpoint was on May 2020, starting from post liver resection, the median follow-up time was 17.4 months (range 6.6-28.7 months) during which 54.9% (50 of 91) of the patients had developed tumor recurrence. The median RFS was 9.7 months (range 0.2-28.7 months), with a 12-month recurrence rate of 44.0% (40 of 91). The liver (n = 37, 74%) was the most common site of recurrence followed by the lung (n = 10, 20%) with infrequent recurrence in lymph nodes (n = 2, 4%) and bone (n = 1, 2%). Single organ recurrences occurred in 47 patients, while three patients developed multiple organ recurrences including two patients with lung and lymph node metastases with the third patient having lung and bone metastases.

As presented in Figure S1, ctDNA analysis was applied to 271 blood samples, including 53 samples in the baseline, 65 samples in pre-operative, 82 samples in post-operative and 49 samples in post-ACT and 22 samples at PD. A total of 84 cases received pre-operative chemotherapy. 7 patients treated without preoperative chemotherapy before surgery had limited liver metastasis and a low clinical risk score. The patients' clinical characteristics at the four different time points are provided in Table S2. The pre-operative blood samples were collected with a median time of one day before liver resection; post-operative blood samples were obtained with a median time of 38 days after liver resection prior to adjuvant therapy; post-ACT blood samples were obtained with a median time of 8 days after the completion of adjuvant chemotherapy, and the PD blood samples were obtained with a median time of 10 days after disease progression was detected by computed tomography (CT) scan. The whole-exome sequencing (WES) analysis was applied to 50 primary colorectal specimens obtained from biopsy (n = 18) and surgery (n = 32), as well as 48 patient-matched liver metastases specimens obtained following hepatic metastasectomy.

### Molecular alterations in matched tissue and blood

Fifty matched primary tumors, and baseline ctDNA were available for the consistency analysis of tissue and blood. 326 mutations spanning 157 genes from the primary tumor and 226 mutations spanning 106 genes from the baseline ctDNA were identified with the 451-gene panel. Furthermore, 121 common mutations from 45 genes were detected in paired tumor tissue and ctDNA samples, accounting for 28.1% of all the mutations.

**Figure 1:**
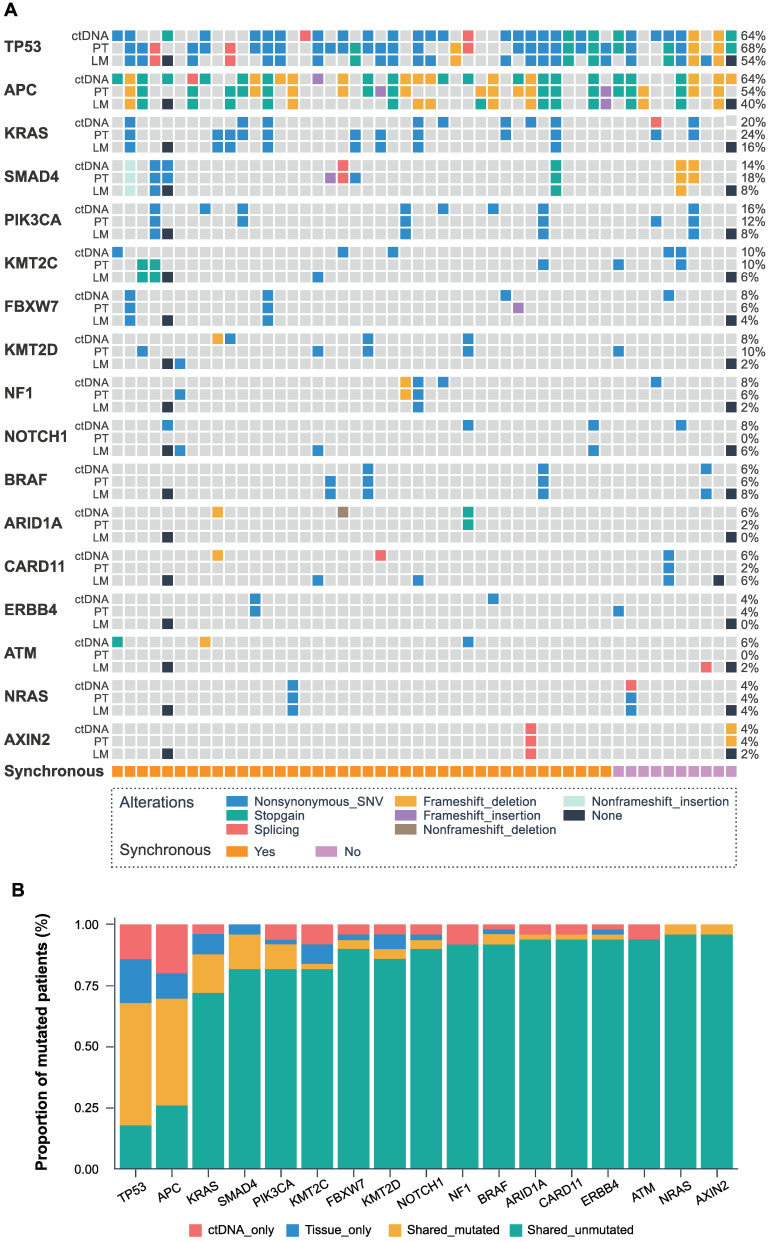
** The landscape and consistency of tumor tissue and baseline blood in 50 patients.** (A) Genomic alterations detected from baseline ctDNA, primary tumor (PT), and liver metastases (LM). The different colors represent different mutant types. The black dot represents the lack of two colorectal liver metastases cases. Each column represents a patient, and each row represents a gene. The sidebars represent the mutation rate of the 50 patients in our study. The lowest pillars represent the clinical characteristics of synchronous or metachronous liver metastases. (B) The mutation consistency (shared mutated and shared unmutated) of 50 matched primary tissues and baseline blood.

The identified genes with high mutation frequency are presented in Figure 1A. The most common somatic variants in primary and liver metastatic tissues were *TP53*, *APC*, and *KRAS*, while the most frequent mutated genes in baseline ctDNA were also *TP53* (64%), *APC* (64%) and *KRAS* (20%), with *SMAD4* (14%), *PIK3CA* (16%), *KMT2C* (10%), *FBXW7* (8%), *KMT2D* (8%), *NF1* (8%), *BRAF* (6%) and *NRAS* (4%) genes also expressing high mutation frequency. The matched primary tissue and blood samples had a shared mutated and unmutated consistency rate of 68.0% in *TP53*, 70.0% in *APC*, 88.0% in *KRAS*, 96.0% in *SMAD4*, 92.0% in *PIK3CA*, 84.0% in *KMT2C*, 94.0% in *FBXW7*, 90.0% in *KMT2D*, 94.0% in *NF1,* 96.0% in *BRAF* and 100% in *NRAS*, as shown in Figure 1B. Statistical analysis showed that all the 451 genes had no significant difference between the ctDNA and the tumor tissue by McNemar's test (*P* > 0.05).

### Dynamic changes of ctDNA during the treatment of CRLM

**Figure 2:**
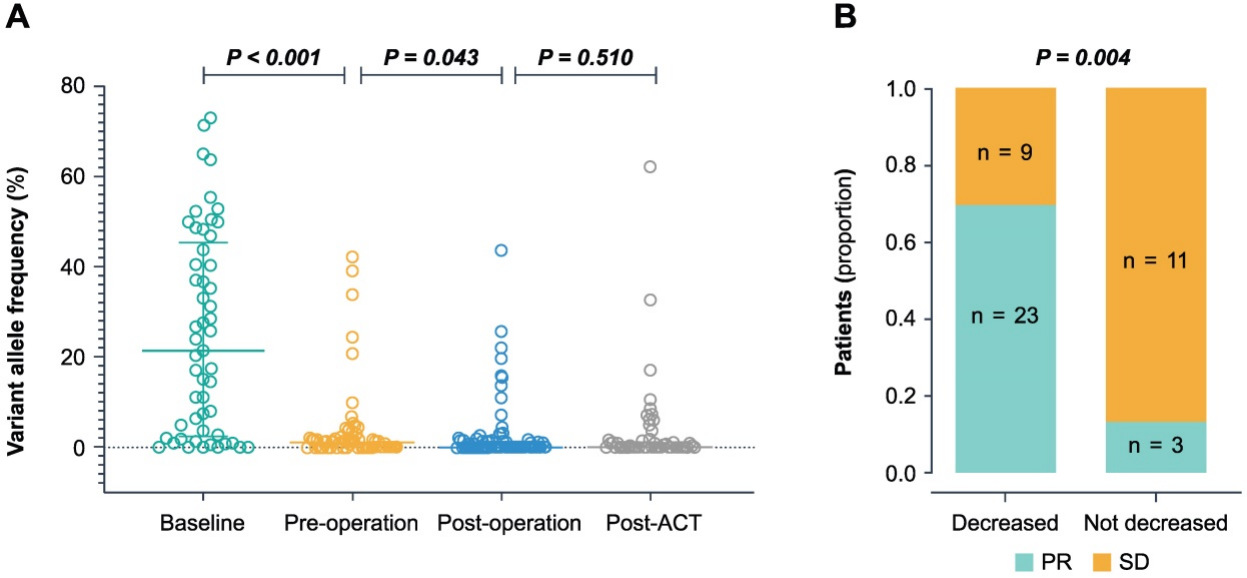
** The dynamic changes of the ctDNA level during treatment.** (A) The ctDNA VAF in baseline, pre-operation, post-operation, and post-ACT. *P*-value was calculated using the Wilcoxon rank-sum test. (B) Association between decreased ctDNA VAF during the pre-operative chemotherapy and the tumor response. The decreased group represents a decrease of more than 10-fold, and the not decreased group represents an increase or a decrease of less than 10-fold. PR partial response, SD stable disease.

Next, we sought to analyze the dynamic changes of ctDNA in four different time points during CRLM therapy (Figure 2A). The median VAF in baseline ctDNA was 21.42% (range 0-72.94%), with a 88.7% (47/53) positive detection rate. The level of baseline ctDNA was significantly correlated with the diameter of liver metastases (*P* < 0.001) and CRS (*P* = 0.008) (Table S3).

During pre-operative chemotherapy, the ctDNA level significantly decreased to a median VAF of 1.04% (range 0-42.19%, *P* < 0.001) prior to surgery. In the 46 patients who had baseline ctDNA level, preoperative ctDNA level and tumor response after neo-adjuvant chemotherapy, we found that a 10 fold decrease in VAF predicted a significantly better tumor response (*P* = 0.004) (Figure 2B). After liver resection, the level of ctDNA declined significantly compared to the pre-operative setting (*P* = 0.043) (Figure 2A). The median VAF decreased to 0% post-operatively (range 0-43.61%) with a positive rate of 41.0% (34 of 83 patients). The post-operative ctDNA-positive patients showed higher pre-operative ctDNA VAF (median 1.52%, range 0-42.19%, n = 22) compared to ctDNA-negative group (median 0%, range 0-24.4%, n = 36) (*P* = 0.0075). Following post-ACT, the ctDNA positive rate was 44.9% (22 of 49). After disease progression, the ctDNA positive rate was 95.5% (21 of 22), and the ctDNA VAF (median 6.08%, range 0-62.13%, n = 22) increased significantly compared to the post-ACT setting (*P* = 0.016).

### Serial ctDNA detection and the survival of patients with CRLM

**Figure 3:**
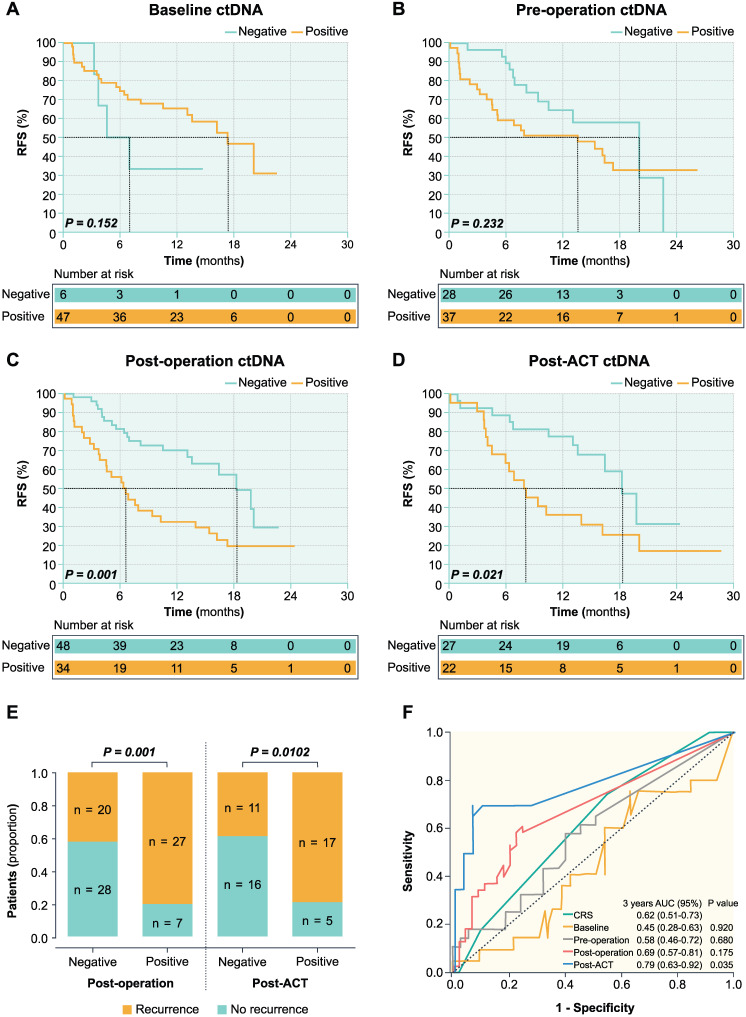
** Prognostic value of serial ctDNA in patients with CRLM.** Kaplan-Meier survival analysis shows the probability of recurrence-free survival (RFS) stratified by ctDNA status of baseline (A), pre-operation (B), post-operation (C), and post-ACT (D). (E) Dichotomized association between disease recurrence and ctDNA status in the post-operative and post-ACT setting. (F) Time-dependent ROC curves of survival prediction between the CRS and VAF of ctDNA at four-time points.

In the post-operative setting, the overall recurrence rate was 41.7% (20 of 48 patients) in ctDNA-negative patients and 79.4% (27 of 34 patients) in ctDNA-positive patients (Figure 3E). The ctDNA-negative patients had significantly longer RFS than those with ctDNA-positive (HR 0.374; 95% CI, 0.205-0.682; *P* = 0.001) (Figure 3C). In the post-ACT setting, the overall recurrence rate was 40.7% (11 of 27 patients) in ctDNA-negative patients and 77.3% (17 of 22 patients) in ctDNA-positive patients (Figure 3E). The ctDNA-negative patients had significantly longer RFS compared to those with positive post-ACT ctDNA (HR 0.406; 95% CI, 0.189-0.873; *P* = 0.021) (Figure 3D).

As shown in Figure 3 and Table 1, post-operative (*P* = 0.001) and post-ACT ctDNA (*P* = 0.021) status remained an independent predictor of RFS after adjusting for known clinicopathological risk factors. However, ctDNA status in baseline (*P* = 0.152), pre-operative (*P* = 0.232), and changes after pre-operative chemotherapy were not significantly associated with the RFS. In post-operative setting, the 6-month recurrence rate was 18.6% (9 of 48 patients) in ctDNA-negative patients and 55.9% (19 of 34 patients) in ctDNA-positive patients, while the 9-month recurrence rate was 41.7% (20 of 48 patients) in ctDNA-negative patients and 70.6% (24 of 34 patients) in ctDNA-positive patients (Figure 3E). In post-ACT setting, the 6-month recurrence rate was 14.8% (4 of 27 patients) in ctDNA-negative patients and 36.4% (8 of 22 patients) in ctDNA-positive patients, while the 9-month recurrence rate was 22.2% (6 of 27 patients) in ctDNA-negative patients and 54.5% (12 of 22 patients) in ctDNA-positive patients. In the post-ACT setting, 4 patients had single lung metastases among the 11 patients with progression in ctDNA negative patients. While in ctDNA positive patients, 1 patient had single lung metastases and 2 patients developed multiple organ recurrences (one patient with lung and lymph node metastases and another patient having lung and bone metastases) among the 17 patients with progression. The proportion of single lung metastases was significantly higher in ctDNA negative patients than ctDNA positive patients.

Furthermore, time-dependent ROC curves were used to compare the sensitivity and specificity of survival prediction, including the CRS and the VAF of ctDNA (continuous variable) at four time points (Figure 3F). In the ROC analysis for RFS prediction, ctDNA in the post-ACT setting with an area under the ROC curve (AUROC) of 0.79 (95% CI 0.63-0.92, *P* = 0.035) had a better predictive significance compared to the CRS and post-operative ctDNA status (Figure 3F).

### The association between ctDNA dynamic changes during adjuvant chemotherapy and RFS

**Figure 4:**
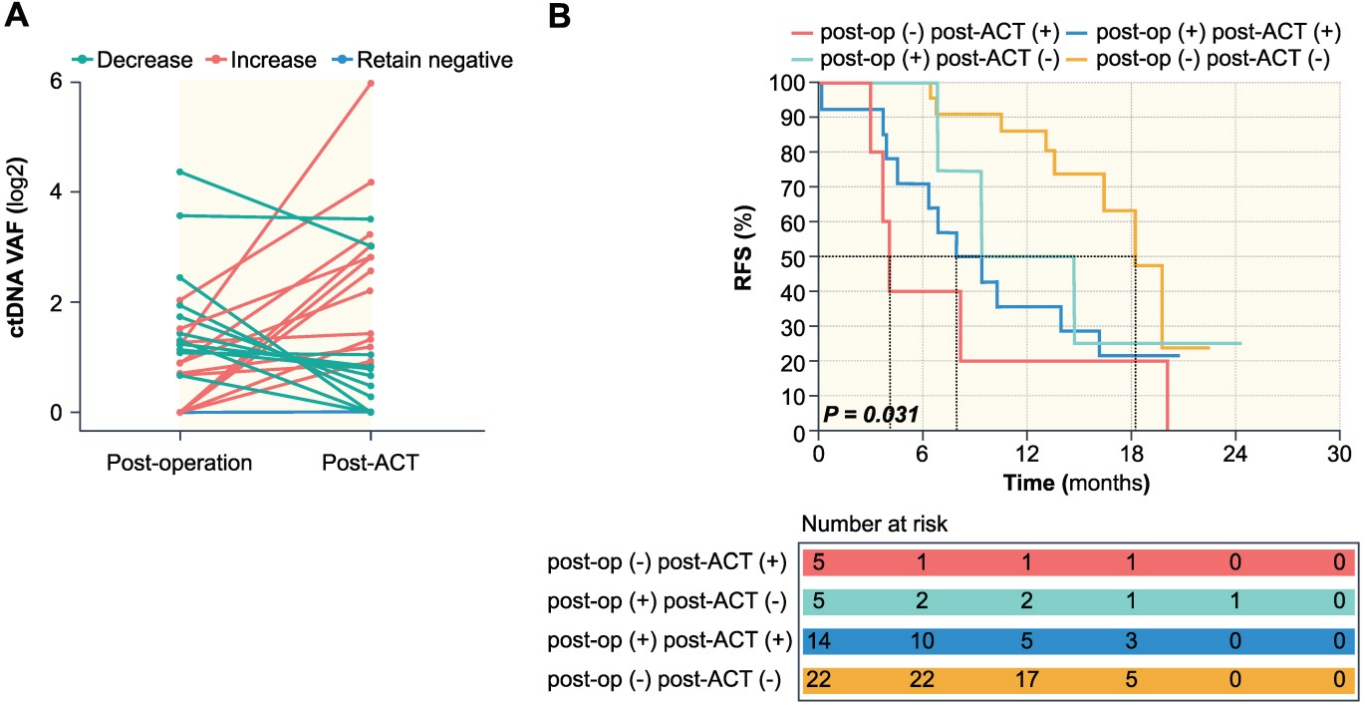
** The association of dynamic changes of ctDNA during adjuvant chemotherapy with RFS.** (A) ctDNA dynamic changes from post-operation to post-ACT. (B) Kaplan-Meier survival analysis of RFS for 46 patients stratified by a combination of post-operative and post-ACT ctDNA status. *P* = 0.031 for statistical comparison between the four groups.

During adjuvant chemotherapy, the ctDNA level of 22 patients remained negative, whereas 24 patients experienced dynamic changes, among which 11 patients showed a decrease while 13 patients showed an increase (Figure 4A). Patients with decreased ctDNA VAF after adjuvant chemotherapy had a recurrence rate of 63.6% (7 of 11 patients), compared to 92.3% (12 of 13 patients) in patients with increased ctDNA VAF. Patients were then stratified using a combination of post-operative and post-ACT ctDNA status, and further prognostic analysis was done based on ctDNA clearance following postoperative adjuvant chemotherapy. The negative to positive group (n = 5) had a recurrence rate of 100% with a median RFS of 4.1 months (range 3-20.1 months). The positive to positive group (n = 14) had a recurrence rate of 78.6% (11/14) with a median RFS of 7.9 months (range 0.2-20.8 months), while post-operative ctDNA change from positive to negative (n = 5) had a recurrence rate of 60.0% (3/5) with a median RFS of 14.7 months (0.9-24.4 months), and the negative to negative group (n = 22) had a recurrence rate 36.4% (8/22) with a median RFS of 14.6 months (range 6.4-22.5 months). Kaplan-Meier survival analysis showed statistical significance between the four groups (*P* = 0.031) (Figure 4B).

## Discussion

In this study, our results demonstrated that serial analysis of ctDNA status during the treatment of the CRLM patient has three main clinical utilities: firstly, dynamic changes in ctDNA levels induced by pre-operative chemotherapy can predict tumor response. Secondly, post-operative and post-ACT ctDNA levels can reflect the MRD and predict recurrence after hepatectomy. Finally, dynamic changes in ctDNA during MRD monitoring might help to determine adjuvant management decisions. Compared to previous studies on ctDNA analysis in CRLM undergoing liver resection, we used a more systematic ctDNA sampling approach and included more patients in this study.

Previous studies have demonstrated excellent concordance between RAS status in plasma and tumor tissue from patients with primary colorectal cancer and liver metastases 21. Consistent with these results, our data indicated high consistency between the detected molecular alterations in the baseline ctDNA and primary and liver metastases tissue. Among these, the consistency of TP53 and APC genes is slightly lower, thus using a multigene panel-based testing might improve the sensitivity of ctDNA detection. We hypothesize that serial ctDNA detection using an NGS panel-based approach in our study can be used to less-invasively track changes in the genetic composition of cellularly and molecularly heterogeneous tumors and act as a biomarker for the prognosis and treatment stratification of CRLM.

In this study, a higher level of baseline ctDNA was significantly correlated with higher tumor burden, such as more extensive liver metastases and higher CRS. In addition, our results showed that a significant decrease in pre-operative ctDNA level during the pre-operative chemotherapy could predict the better tumor response rate, suggesting that dynamic ctDNA monitoring might assist in tailoring the intensity of pre-operative therapy. Nevertheless, our current study did not show that persistently detectable pre-operative ctDNA was associated with shorter post-operative survival, which is different from previous studies which reported that preoperative ctDNA testing helps select patients suitable for liver metastasectomy 19. We believe that although the patient's tumor burden has decreased after preoperative chemotherapy, liver metastases and/or primary tumors still exist, so the level of plasma ctDNA in patients before surgery cannot accurately predict the prognosis of patients.

Following surgery with curative intent or adjuvant chemotherapy, detection of ctDNA may signal the presence of MRD even in the absence of any other clinical or imaging evidence of recurrence. In patients with stage III colon cancer and locally advanced rectal cancer undergoing radical resection, several studies have reported that ctDNA testing can identify MRD and identify patients with a higher risk of recurrence 17, 18. However, ctDNA analysis remains scarcely utilized in CRLM patients undergoing hepatectomy. Our result shows that post-operative ctDNA status is an independent predictor of RFS after adjusting for known clinicopathological risk factors. This is consistent with Michael J. Overman et al.'s study submitted to the Journal of Clinical Oncology 35, no. 15_suppl (May 20, 2017) 3522-3522, which reported that postoperative ctDNA is associated with RFS (*P* = 0.002) in 43 CRLM patients who had all visible diseases removed, with a 2-year RFS of 0 % vs 47% in ctDNA positive and ctDNA negative patients respectively. In addition, our study showed that post-ACT ctDNA was associated with significantly shorter RFS after hepatectomy. Moreover, the prognostic discriminatory capacity of ctDNA in post-ACT was superior to that of post-operative ctDNA measurement. To our knowledge, this has not been reported in patients with CRLM after liver resection yet. However, in stage III colon cancer patients, results have shown that post-chemotherapy ctDNA detection can define a subset of patients with a higher risk of recurrence despite completion of adjuvant treatment 15.

Adjuvant chemotherapy administered after resection of CRLM may reduce the risk of recurrence and improve survival, but its benefit remains controversial. Two phase III trials (FFCD trial 9002 and the ENG trial) both showed a nonsignificant trend for improvement in DFS and OS for patients treated with adjuvant chemotherapy 22, 23, but both trials close prematurely because of slow accrual. The EORTC Intergroup trial 40983 showed that perioperative chemotherapy with FOLFOX4 improved DFS in patients with initially resectable CRLM 24, but no difference in OS 25. Recently, the JCOG0603 study found that postoperative chemotherapy with mFOLFOX6 improves DFS but worsens OS over surgery alone for the patients with CRLM. These findings suggest that part of CRLM patients cannot benefit from postoperative adjuvant chemotherapy after hepatectomy. At present, several studies are exploring personalized approaches that use ctDNA analysis to guide the initiation and modification of adjuvant chemotherapy. In the IDEA-FRANCE Trail, ctDNA analysis of patients with stage III colon cancer after surgery and before adjuvant chemotherapy showed that ctDNA was not only of prognostic value, but also of predictive value for a treatment duration of 3 or 6 months. However, not all patients can achieve ctDNA clearance after adjuvant chemotherapy. Jeanne Tie reported that ctDNA status changed from negative to positive after chemotherapy in 6 of 62 patients (9.7%) after 3 months of chemotherapy; and in 5 of 53 patients (9.4%) at chemotherapy completion. In patients with positive postoperative ctDNA, a positive ctDNA after chemotherapy was associated with an inferior RFI compared with patients in whom ctDNA became undetectable after chemotherapy (HR, 3.7; *P* = 0.04). Conversely, ctDNA status changed from positive to negative in 9 of 16 patients (56.3%) after 3 months of chemotherapy; and 8 of 13 patients (61.5%) at chemotherapy completion. In patients with negative postoperative ctDNA, a negative ctDNA result after chemotherapy was associated with a superior RFI compared with patients in whom ctDNA became detectable after chemotherapy (HR, 6.5; *P* < 0.001) 15. In our present study, results show that the ctDNA level of 22 patients remained negative, whereas 24 patients experienced dynamic changes, among which 11 patients showed a decrease while 13 patients showed an increase during adjuvant chemotherapy. Patients with decreased ctDNA VAF after adjuvant chemotherapy had a recurrence rate of 63.6% (7 of 11 patients), compared to 92.3% (12 of 13 patients) in patients with increased ctDNA VAF. This result suggests that serial analysis of ctDNA in CRLM after hepatectomy could potentially be used as a real-time marker to determine the subgroups of patients who would or would not benefit from adjuvant chemotherapy. Further clinical trials are needed to establish whether prolong treatment duration or a shift to a second-line regimen can improve survival in patients with detectable post-ACT ctDNA.

## Limitations

This study has several limitations. Firstly, the OS data in this study are not mature, a long term follow-up is needed to confirm our results. Secondly, this is a single-center, small cohort study, thus larger multicenter studies are needed to validate our findings further. Thirdly, not all patients provided serial plasma samples at the different time points set during CRLM therapy for ctDNA analysis. This relatively small sample size may influence the statistical power. Finally, our data used ctDNA VAF to reflect tumor status, however, no consensus was available regarding the clinically relevant cut-offs and thresholds for categorizing ctDNA levels (continuous data). A more standardized method for quantifying ctDNA levels needs to be established for further research.

## Conclusions

Our study demonstrates that noninvasive molecular profiling using ctDNA analysis can predict the clinical outcome in patients with CRLM. We envision that dynamic ctDNA analysis, especially in a post-ACT setting, can be used to not only reflect MRD but also to determine rational personalized adjuvant therapy after the resection of CRLM. We encourage the incorporation of ctDNA into future clinical trials as a biomarker for the treatment stratification of patients with CRLM.

## Supplementary Material

Supplementary figures and tables.

## Figures and Tables

**Table 1:** Univariate and multivariate analysis of known clinicopathological risk factors and post-ACT ctDNA associated with RFS (n = 49)

Variables	Univariate			Multivariate		
	HR	95%CI	*P*-value	HR	95%CI	*P*-value
Age, years (< 60/ ≥ 60)	1.527	0.652-3.575	0.329			NA
Gender (female/male)	1.767	0.781-4.002	0.172			NA
Primary tumor (Right/Left)	0.865	0.279-2.679	0.801			NA
Nodal involvement of primary tumor (No/Yes)	0.614	0.283-1.336	0.219			NA
Time between primary tumor and liver metastases (< 12/ ≥ 12months)	0.766	0.293-2.003	0.587			NA
Diameter of the largest LM (< 5/ ≥ 5cm)	1.412	0.620-3.219	0.412			NA
Preoperative CEA level (< 5/ ≥ 5ng / mL)	1.152	0.446-2.977	0.770			NA
Number of LM (< 2/ ≥ 2)	0.476	0.211-1.075	0.074			NA
CRS (0-2/3-5)	0.656	0.311-1.382	0.267			NA
Preoperative chemotherapy (Yes/No)	1.936	0.684-5.479	0.211			NA
Postoperative chemotherapy (Yes/No)	0.072	0.005-1.022	0.0519			NA
Concomitant ablation (Yes/No)	1.186	0.485-2.902	0.709			NA
KRAS (mt-/mt+)	0.944	0.418-2.130	0.889			NA
BRAF (mt-/mt+)	1.248	0.207-7.520	0.809			NA
Post-ACT ctDNA (Negative/Postive, n = 49)	**0.406**	**0.189-0.873**	**0.021**	**0.417**	**0.194-0.896**	**0.025**

HR greater and less than 1 indicates increased and decreased relapse risk, respectively. HR: hazard ratio; RFS: recurrence-free survival; CRS: clinical risk score; post-ACT: postoperative adjuvant chemotherapy; ctDNA: circulating tumor DNA.

## Abbreviations

ctDNA: circulating tumor DNA
CRLM: colorectal liver metastases
DFS: disease-free survival
mCRC: metastatic colorectal cancer
LM: liver metastases
m-CS: modified clinical score
MRD: minimal residual disease
NGS: next-generation sequencing
OS: overall survival
post-ACT: post-operative adjuvant chemotherapy
RFS: recurrence-free survival
VAF: variant allele frequency
WES: whole-exome sequencing
