# Supplementary material for: Dynamic monitoring of circulating tumor DNA to predict prognosis and efficacy of adjuvant chemotherapy after resection of colorectal liver metastases
Source: Theranostics. 2021 May 12;11(14):7018–28. doi: 10.7150/thno.59644 (PMC8171084; doi:10.7150/thno.59644)
Supplement: Supplementary file 1 — Supplementary figures and tables. [file thnov11p7018s1.pdf]

Supplementary Table 1. The clinical characteristics of patients with CRLM in this study.

**Characteristics**	**(n = 91)**
Age at liver resection（years）	Median 59 (34 - 66)
Gender	
Male	47 (51.6%)
Female	44 (48.4%)
Primary tumor location	
Right-sided	18 (19.8%)
Left-sided	73 (80.2%)
Nodal involvement of primary tumor	
N1-2	59 (64.8%)
N0	32 (35.2%)
Time between primary tumor and liver metastases	
Synchronous	71 (78.0%)
Metachronous	20 (22.0%)
Preoperative CEA (ng/mL)	
≤ 5	20 (22.0%)
> 5	71 (78.0%)
Preoperative chemotherapy	
Chemotherapy alone	47 (51.6%)
Chemotherapy + Cetuximab	23 (25.3%)
Chemotherapy + Bevacizumab	14 (15.4%)
No preoperative chemotherapy	7 (7.7%)
Best Response before surgery	
PR	43 (47.3%)
SD	41(45.1%)
NA	7 (7.7%)

Postoperative chemotherapy	
Chemotherapy alone	65 (71.4%)
Chemotherapy + Cetuximab	15 (16.5%)
Chemotherapy +Bevacizumab	3 (3.3%)
No postoperative chemotherapy	8 (8.8%)
Concomitant ablation	
Yes	27 (29.7%)
No	64 (70.3%)
Number of liver metastasis	
= 1	25 (27.5%)
> 1	66 (72.5%)
Extent of liver metastases	
Unilateral	40 (44.0%)
Bilateral	51 (56.0%)
Diameter of the largest liver metastasis (cm)	Median 4.3 (1.0 – 15.7)
CRS	
Low risk (Score 0-2)	33 (36.3%)
High risk (Score 3-5)	58 (63.7%)

CRLM, colorectal liver metastasis

CEA, carcinoembryonic antigen

PR, partial response

SD, stable disease

CRS, clinical risk score

Supplementary Table 2. The clinical characteristics of patients with CRLM at four time points.

Characteristics	Baseline ctDNA (n = 53)	Pre-operation ctDNA (n = 65)	Post-operation ctDNA (n = 82)	Post-ACT ctDNA (n = 49)
Age at liver resection（years）	Median 56 (20 - 73)	Median 56 (20 - 73)	Median 56 (20 - 75)	Median 56 (20 - 73)
Gender				
Male	32 (60.4%)	43 (66.2%)	53 (64.6%)	30 (61.2%)
Female	21 (39.6%)	22 (33.8%)	29 (35.4%)	19 (38.8%)
Primary tumor location				
Right-sided	9 (17.0%)	11 (16.9%)	16 (19.5%)	9 (18.4%)
Left-sided	44 (83.0%)	54 (83.1%)	66 (80.5%)	40 (81.6%)
Nodal involvement of primary tumor				
N1-2	34 (64.2%)	43 (66.2%)	57 (69.5%)	32 (65.3%)
N0	19 (35.8%)	22 (33.8%)	25 (30.5%)	17 (34.7%)
Prior resection of the primary tumor				
No	33 (62.3%)	36 (55.4%)	49 (59.8%)	31 (63.3%)
Yes	20 (37.7%)	29 (44.6%)	33 (40.2%)	18 (36.7%)
Time between primary tumor and liver metastases				
Synchronous	42 (79.2%)	53 (81.5%)	66 (80.5%)	41 (83.7%)
Metachronous	11 (20.8%)	12 (18.5%)	16 (19.5%)	8 (16.3%)
Preoperative CEA (ng/mL)				
≤ 5	22 (41.5%)	27 (41.5%)	32 (39.0%)	22 (44.9%)
> 5	31 (58.5%)	38 (58.5%)	50 (61.0%)	27 (55.1%)
Preoperative chemotherapy				
Yes	51 (96.2%)	64 (98.5%)	76 (92.7%)	44 (89.8%)
No	2 (3.8%)	1 (1.5%)	6 (7.3%)	5 (10.2%)
Extend of liver metastases				
Solitary	18 (34.0%)	18 (27.7%)	23 (28.0%)	11 (22.4%)
Multifocal	35 (66.0%)	47 (72.3%)	59 (72.0%)	38 (77.6%)
Unilateral	27 (50.9%)	28 (43.1%)	36 (43.9%)	20 (40.8%)

Bilateral	26 (49.1%)	37 (56.9%)	46 (56.1%)	29 (59.2%)
Concomitant ablation				
Yes	39 (73.6%)	20 (30.8%)	25 (30.5%)	11 (22.4)
No	14 (26.4%)	45 (69.2%)	57 (69.5%)	38 (77.6%)
Postoperative chemotherapy				
Yes	47 (88.7%)	58 (89.2%)	76 (92.7%)	47 (95.9%)
No	6 (11.3%)	7 (10.8%)	6 (7.3%)	2 (4.1%)
CRS				
Low risk (Score 0-2)	21 (39.6%)	18 (27.7%)	30 (36.6%)	21 (42.9%)
High risk (Score 3-5)	32 (60.4%)	47 (72.3%)	52 (63.4%)	28 (57.1%)

CRLM, colorectal liver metastases

CEA, carcinoembryonic antigen

CRS, clinical risk score

Supplementary Table 3. Relationship of ctDNA status and clinical characteristics of CRLM.

Variables	Baseline ctDNA (n = 53)			Pre-operation ctDNA (n = 65)			Post-operation ctDNA (n = 82)			Post-ACT ctDNA (n = 49)		
	**Low level (n = 26)**	**High level (n = 27)**	***P***	**Negative (n = 28)**	**Positive (n = 37)**	***P***	**Negative (n = 48)**	**Positive (n = 34)**	***P***	**Negative (n = 27)**	**Positive (n = 22)**	***P***
Age at liver resection (years)			0.928			0.156			0.443			0.553
< 60	18	19		22	23		33	26		19	18	
≥ 60	8	8		6	14		15	8		8	4	
Gender			0.340			0.420			0.631			0.386
Female	12	9		11	11		18	11		9	10	
Male	14	18		17	26		30	23		18	12	
Primary tumor location			0.409			0.318			0.191			0.598
Right-sided	21	24		26	30		42	26		22	20	
Left-sided	5	3		2	7		6	8		5	2	
Nodal involvement of primary tumor			**0.013**			0.182			0.249			0.325
No	5	14		12	10		17	8		11	6	
Yes	21	13		16	27		31	26		16	16	
Disease free interval (DFI, months)			0.947			0.861			0.476			0.943
> 12	5	5		5	6		7	7		5	3	
< 12	21	22		23	31		41	27		22	19	
Diameter of the largest LM (cm)			**<0.001**			0.388			0.354			0.586
< 5	23	9		16	25		33	20		19	17	
≥ 5	3	18		12	12		15	14		8	5	
CEA level (ng/mL)			0.280			0.246			0.272			0.390
< 5	3	1		3	8		7	8		3	5	
≥ 5	23	26		25	29		41	25		24	15	
Number of liver metastasis			0.497			0.700			0.443			0.207

< 2	10	8		8	9		15	8		9	3	
≥ 2	16	19		20	28		33	26		18	19	
CRS			**0.008**			0.069			0.503			0.407
0-2	15	6		11	7		19	11		13	8	
3-5	11	21		17	30		29	23		14	14	
Recurrence			0.132			0.122			< **0.001**			**0.010**
No	11	17		16	14		28	7		16	5	
Yes	15	10		12	23		20	27		11	17	

Low level and high level respectively represented lower-than-median and higher-than-median VAF of baseline ctDNA.

ctDNA, circulating tumor DNA

CRLM, colorectal liver metastasis

LM, liver metastasis

CEA, carcinoembryonic antigen

CRS, clinical risk score

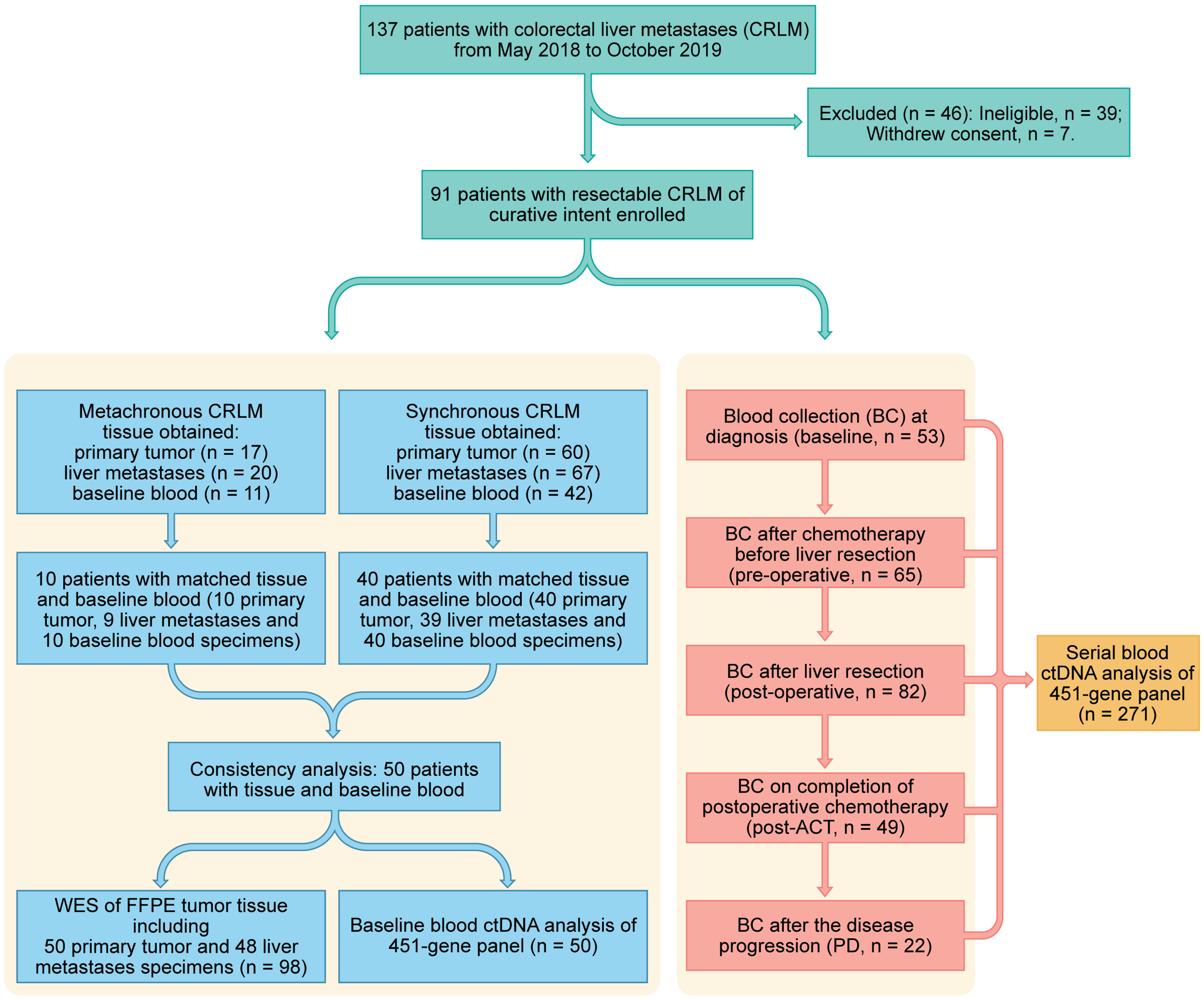

Supplemental Figure 1. Flow chart of patients enrolled and sample collection at different time points for analysis.
